# *Habrobracon hebetor* and *Pteromalus cerealellae* as Tools in Post-Harvest Integrated Pest Management

**DOI:** 10.3390/insects10040085

**Published:** 2019-03-27

**Authors:** George N. Mbata, Sanower Warsi

**Affiliations:** Agricultural Research Station, Fort Valley State University, Fort Valley, GA 31030, USA; Sanowerw@yahoo.com

**Keywords:** pesticide residue, post-harvest pest, parasitoid, entomopathogens, commodities

## Abstract

Consumers are increasingly demanding pesticide-free grain/legumes and processed foods. Additionally, there are more restrictions, or complete loss, of insecticides labelled for use in managing stored grain insects in post-harvest ecosystems. Suppression of post-harvest pests using parasitic wasps is a more sustainable alternative than chemical pesticides. *Habrobracon hebetor* (Say) (Hymenoptera: Braconidae) and *Pteromalus cerealellae* Ashmead (Hymenoptera: Pteromalidae) are two important parasitoids that limit economically important pests of stored products. Host searching ability and reproductive performances of *H. hebetor* and *P. cerealellae* depend on a wide range of factors, such as host species, commodities, and environmental conditions. Further, use of entomopathogens can complement the ability of parasitoids to regulate pest populations. This review provides information on aspects of *H. hebetor* and *P. cerealellae* biology and successful regulation of post-harvest pest populations.

## 1. Introduction

Stored product pests cause severe economic losses due to the infestation of commodities in stored grain ecosystems including silos, bakeries, food processing industries, flourmills, and pet food factories. Stored grain managers rely substantially on strategies involving the application of synthetic insecticides to manage stored product pests [1,2]. Although insecticides can be effective, their repeated and indiscriminative use will cause insecticide resistance and detrimental non-target effects, potentially leading to the loss of biodiversity [3]. Insecticide use patterns in post-harvest integrated pest management (IPM) could expose farmers and commodity warehouse workers to acute or chronic exposure to fumigant volatiles [4]. Additionally, consumers could potentially be exposed to pesticide residues in foods [4]. Furthermore, restrictions on insecticide use patterns and increasing consumer demand for pesticide-free foods are catalyzing the search for safe, non-toxic, and sustainable pest management strategies in post-harvest systems [5,6].

Parasitic wasps present an alternative and environmentally compatible approach to overcoming the challenges of synthetic insecticide use in post-harvest systems. Parasitoids do not negatively affect the environment, humans or beneficial organisms. These natural enemies can reproduce continuously for as long as the hosts or alternative hosts are available, thus ensuring sustainability of their populations for long-term regulation of pest populations [7]. Moreover, released parasitoid wasps have the ability to disperse very quickly and locate hosts in hidden corners and crevices in the storage structures [8,9]. In addition, potential allergens including insect exuviae and fragments are significantly reduced when parasitoids are used compared with other IPM strategies [10]. The parasitoids *Habrobracon hebetor* (Say) (Hymenoptera: Braconidae) and *Pteromalus cerealellae* Ashmead (Hymenoptera: Pteromalidae) are among the important ectoparasitoids of stored product moths and beetles, respectively.

This paper examines the distribution, life history, host range, behavior, and mode of parasitism of the two parasitoids. The efficacies of the parasitoids in laboratory experiments and field trials were also reviewed. The information provided here will assist in designing IPM programs utilizing biological control.

## 2. Distribution

*Habrobracon hebetor* and *P. cerealellae* are cosmopolitan in distribution [11,12]. The taxonomy of *H. hebetor* has been revised several times and this species is also known under the following synonyms: *Bracon hebetor* Say, *Bracon juglandis* Ashmead, and *Hebrabracon junglandis* Ashmead [13]. In the older literature this wasp is commonly called *B. hebetor*. The post-harvest populations of *H. hebetor* fluctuate throughout the year [14,15]. While environmental conditions in spring are not particularly conducive to *H. hebetor*, high summer temperatures favor population increases, and wasp populations generally reach maximum numbers in autumn.

*Pteromalus cerealellae* (Ashmead) has other synonyms including *Habroaytus cerealellae* (Ashmead 1902) and *Pteromalus semota* (Walker 1834) [16]. Documented distribution of *P. cerealellae* extends to Africa, Asia, Caribbean and Europe and North America [16].

## 3. Life History and Mating Behavior of *Habrobracon hebetor* and *Pteromalus cerealellae*

*Habrobracon hebetor* is a small parasitic wasp that is only 2 mm in length, weighs less than 1 mg, is known to have cryptic sibling species that attacks the crop pest *Helicoverpa armigera* (Hübner), and completes its development in about 9 days when reared on *Helicoverpa armigera* (Hübner) at 25 ± 2 °C, 65 ± 5% RH, and 14:10 (L:D) [17,18,19]. Female wasps preferably parasitize fourth or fifth instars of moth hosts for egg laying. At 28 °C, female *H. hebetor* lays 17.46 and 9.64 eggs per day on diapausing and non-diapausing larvae of *Plodia interpunctella,* respectively [20]. Eggs (0.52 mm length and 0.12 mm width) of *H. hebetor* are hymenopteriform in shape and are typically attached to paralyzed host larvae. *H. hebetor* has an extremely short duration of development that lasts only 12 days from egg to adult [21,22]. The last instar (2.64 mm in length, 0.29 mm head capsule, and 0.95 mm width) lasts about 4 days before spinning small white cocoons for pupation [21]. The pupal stage lasts about a week [23]. Free-living adult parasitoids begin to search for host larvae to parasitize and lay eggs on immediately after pupation [24]. The mean number of lifetime adult progeny per female is 173.7 in 22 days, with an offspring sex ratio of 1:1 [25]. Male adults have long antennae with 18–20 cylindrical flagellar segments, while females have shorter antennae and are larger in size [26,27,28].

*Habrobracon hebetor* displays the arrhenotokous haplo-diploid sex determination mechanism [29,30]. Haploid male offspring develop from unfertilized eggs parthenogenetically, while diploid males or females emerge from fertilized eggs [29]. Females that copulate with diploid males have lower fertility and produce more male-biased broods, compared with those copulated to haploid males [29]. Females avoid mating with males of the same brood; further, newly emerged females have the ability to recognize their brood mates [11,29]. *H. hebetor* are synovigenic, displaying increased egg production when provisioned diapausing larval *P. interpunctella* [20]. Female *H. hebetor* reared on non-diapausing larvae mature more eggs after three days, while those reared on diapausing *P. interpunctella* larvae mature more eggs from the second day [20]. In addition, oviposition peaks on the third day when *H. hebetor* are reared on diapausing host larvae and on the fourth day if reared on non-diapausing host larvae [20].

Adults of *H. hebetor* are capable of entering photoperiod-induced diapause if exposed to a 10L:14D light regimen at 17.5 °C or 20 °C [31]. Adult wasps, particularly those that are diapausing, are less susceptible to low temperatures and can survive at −5 °C for up to seven days [31]. Egg and pupal stages of *H. hebetor* had supercooling points below −25 °C, but no pupae survived up to 12 d at −5 °C [32].

The pteromalid ectoparasitoid *P. cerealellae* parasitizes a broad host range of pests that develop inside cereal grains and legumes [33]. Adult females oviposit on the late instar of hosts inside the cereal kernels or legume seeds [33,34]. *P. cerealellae* eggs are also hymenopteriform in shape with dimensions of 0.54 × 0.19 mm [12]. Freshly deposited eggs have a transparent chorion [35] that becomes opaque and white after 24–36 h [35]. Eggs hatch into 13-segmented first instars with a head capsule width that ranges from 0.30 ± 0.02 mm to 0.32 ± 0.01 mm [12]. *P. cerealellae* has four larval stages and the larvae have distinct tusk-shaped mandibles, while the pupae and adults have toothed mandibles [35]. Larvae complete development in about 7 days [12]. Adults start to emerge following completion of pupation in 5 days and male adults emerge first [12]. The developmental period from egg to adult at 30 ± 1 °C, 70 ± 5% RH, and 12:12 (L:D) h photoperiod lasts about 11 and 12 days for males and females, respectively [35]. Adult male and female *P. cerealellae* can be differentiated based on length of the antennae. The females have longer antennae (1328 ± 15.2 µm) than males (1308 ± 21.9 µm) [12].

## 4. Hosts of *Habrobracon hebetor* and *Pteromalus cerealellae*

*Habrobracon hebetor* mainly attacks larvae of Lepidopteran stored product pests including *P. interpunctella*, *Ephestia kuehniella* (Zeller), *Ephestia cautella* (Walker), *Anagasta kuehniella* (Zeller), *Galleria mellonella* (Linnaeus), and *Amyelois transitella* (Walker) [35]. Some strains of *H. hebetor* also attack field pests such as *H. armigera*, *Maruca testulalis* (Geyer), *Spodoptera litura* (Fabricius), and *Earias vittella* (Fabricius) [36].

*Pteromalus cerealellae* is a parasitoid of internally developing insects including *Sitotroga cerealella* (Olivier), *Callosobruchus maculatus* (Fabricius), *Lasioderma serricorne* (Fabricius), *Prostephanus truncatus* (Horn), and *Sitophilus* spp. [34].

## 5. Mode of Parasitism of *Habrobracon hebetor* and *Pteromalus cerealellae*

*Habrobracon hebetor* relies on an insecticidal toxin present in its venom gland for paralyzing the host larvae [17]. The wasp’s venom glands are composed of a single layer of eight elongated cells that are connected to the ovipositor [37]. Adult females inject toxin into host larvae by stinging through the cuticle [23]. In *H. hebetor*, the toxin contains five polypeptides, Brh-I, II, III, IV, and V, with a molecular weight of about 70 kDa [17]. These polypeptides block glutamatergic neuromuscular transmission at the presynaptic nerve terminal, resulting in the death of host larvae within 15 min [38,39,40]. In addition, the venom of *H. hebetor* also affects other physiological activities of paralyzed host larvae including decline in the production of reactive oxygen species, suppression of phenoloxidase activity in host hemolymph, and reduced encapsulation of hemocytes [41].

Only female parasitoids feed on the hemolymph of paralyzed host larvae during oviposition. Male wasps can feed on artificial diets including diluted honey [37]. Larvae of *H. hebetor* feed and develop on the cuticle of the paralyzed host until pupation [41]. Larvae of *H. hebetor* can switch to another paralyzed host larva for feeding upon the depletion of nutrients in the immediate host larva [37].

While laying eggs, *P. cerealellae* females inject venom proteins into the host larvae using a stinging apparatus [34]. Injection of venom into the host results in fatal paralysis, which ultimately causes death of the host [34]. Adult and larval stages of *P. cerealellae* derive nutrients from the host hemolymph that oozes from the punctured integument [34].

## 6. Factors Influencing Host Parasitism and Reproductive Efficacies of *Habrobracon hebetor* and *Pteromalus cerealellae*

### 6.1. Effect of Host Density on Habrobracon hebetor and Pteromalus cerealellae

Numerous laboratory studies have reported the effect of host density on the biology of *H. hebetor* [20,42,43,44]. Generally, parasitism by *H. hebetor* increases with the host densities up to an optimum parasitoid-host ratio [43]. In a previous study by Sanower et al., percentage mortality of non-diapausing host larvae resulting from parasitism by *H. hebetor* decreased beyond an optimum parasitoid-host density ratio, but mortality of diapausing larvae was more than 95% at all host densities investigated [20].

Host density also affects the reproductive efficacy and weight of F_1_ progeny of *H. hebetor*. Egg dispersion by *H. hebetor* on larvae of *P. interpunctella* has been shown to decrease as the host density increased [43]. In a study by Rotary and Gerling, a high host parasitoid ratio increased the percentage egg hatch and adult emergence by *H. hebetor* when reared on *G. mellonella* [45]. In a study by Ghimire and Phillips, progeny numbers of *H. hebetor* parasitizing late instars of *P. interpunctella* were higher at high host densities compared to lower densities [44]. The influence of host density on the sex ratio of *H. hebetor* has also been investigated [44,45,46,47]. A previous study showed that the offspring sex ratio (male/total) increased with decreasing host/parasitoid ratio [47]. In addition, it has been reported that the egg laying potential of the wasp increased with an increase in host density and this resulted in male biased progeny [43,48].

Host density has been shown to strongly influence the host mortality and progeny of *P. cerealellae.* A previous study showed that the mortality of host larvae, *C. maculatus*, increased with host density [49]. The attack response of *P. cerealella* to weevil larvae was best described by a type III functional response using an egg limitation model [50]. In this model, the mean attack rate of the parasitoid was estimated to be 1.7 and the upper limit of the response was 24 weevil larvae per individual parasitoid within a 48-h period [50]. The presence of *P. cerealella* resulted in reduced weight losses of cowpea resulting from weevil infestation [50].

### 6.2. Effect of Host Size on Habrobracon hebetor and Pteromalus cerealellae

Host size is a key factor that influences functional responses of parasitic wasps [51]. *H. hebetor* has shown a ten-fold higher attraction toward larger mature wandering larvae than small young larvae [16]. In addition, *H. hebetor* prefers larger sized host larvae for egg laying [51]. In a previous study by Mbata et al., daily lifetime fecundity of *H. hebetor* was higher on the larger larvae of *A. transitella* (55.00 ± 1.90 mg) and *G. mellonella* (262.78 ± 15.17 mg) compared to smaller *P. interpunctella* larvae (20.15 ± 0.92 mg) [33]. Larger larvae provide better food resources and surface area, which influences oviposition in the wasp [23,36]. Furthermore, nutrient-rich diapausing larvae have higher body masses and provide better resources to *H. hebetor* for egg production [20,52].

Host mortality due to parasitism has been shown to vary linearly with increasing host size [53]. It has been reported that the host size influences the reproductive performances of *P. cerealellae* [54]. Larvae of *Sitotroga cerealella* (Olivier) have been found to be more susceptible to *P. cerealellae* than those of *Sitophilus zeamais* and this was ascribed to the larger size of *S. cerealella* larvae [53]. Weights of male and female offspring of this wasp correlated with the size of host, but the weights of female parasitoid progeny were more influenced by the size of host larvae [53,54].

### 6.3. Effect of Host Species, Physiological State of Host, and Maternal Diet of Parasitoid on Progeny of Habrobracon hebetor and Pteromalus cerealellae

Certain host species enhance reproductive performance of wasps better than others. For example, *H. hebetor* has been shown to perform better on stored product moths, such as *E. kuehniella* and *P. interpunctella*, than on crop pests including *H. armigera* and *Malacosoma disstria* Hübner (Lepidoptera: Lasiocampidae) [36,55].

Smith et al. showed that rearing *P. cerealella* on *S. cerealellae* resulted in higher progeny numbers compared with rearing the parasitoid on *S. zeamais* [53]. *S. cerealellae* was found to be highly susceptible to *P. cerealellae* when parent parasitoids were also reared on *S. cerealellae,* but when parent parasitoids were reared on *S. zeamais*, parasitism of *S. cerealellae* decreased [53].

The physiological state of the host has been shown to affect the fecundity and weight of parasitoid offspring [20]. *H. hebetor* reared on diapausing *P. interpunctella* larvae produced more offspring, which were heavier than offspring from parent parasitoids that were fed on diapausing host larvae.

Progeny production, development, and foraging behavior of *H. hebetor* are strongly affected by the diet of the host [56]. A study on the fecundity, developmental period, longevity of adults, and offspring sex ratio of *H. hebetor* on the larvae of *E. kuehniella* raised on four different diets (rice flour, corn flour, wheat flour, and barley flour) demonstrated that the highest number of F_1_ females was recorded when the host diet was rice flour [56]. However, the total number of adult progeny was significantly higher when *H. hebetor* was reared on the larvae of *E. kuehniella* fed on wheat flour combined with 20% glycerol diet [57]. Conversely, a lower number of progeny was obtained when the host diet was whole-wheat flour only [57].

Supplementing parasitoid hosts with sugar enhanced the survival of *P. cerealellae* and resulted in a female-biased sex ratio of offspring [58]. However, the longevity of female wasps was higher than those of the males, irrespective of whether parent parasitoids were provisioned with sugar supplement or not. Lifetime fecundity of *P. cerealellae* was not affected by diet [58].

### 6.4. Effect of Available Space on Efficiency of Habrobracon hebetor and Pteromalus cerealellae

The results of container size on the efficacy of parasitoids with respect to the mortality of hosts have not been consistent. In a laboratory experiment, the proportion of paralyzed larvae did not vary remarkably in response to container size (0.0001672 to 0.00038 m^3^), and more than 90% of host larvae (diapausing and non-diapausing larvae of *P. interpunctella*) were parasitized by *H. hebetor* [59]. However, parasitization of *E. kuehniella* decreased as the volume of storage structures scaled up from 0.006415 to 8 m^3^ [60]. In another study, the mortality of *P. interpunctella* larvae exposed to *H. hebetor* in a cage (0.0029 m^3^) and a storage house (25–32 m^3^) also demonstrated that the mortality of hosts decreased as available storage space increased [61].

In a previous study by Mbata et al., mortality of *C. maculatus* larvae due to parasitism by *P. cerealellae* was significantly higher in small containers of about 5 cm^3^ compared with larger containers [49]. However, mortality was not significantly different in containers that ranged in size from 57.4 to 202.4 cm^3^ [49]. The spacing between seeds likely played a role in the differences in mortality [49]. It is probable that different parasitoids will have different search distances, and this is likely to influence parasitoid efficacy in large storage structures. The numbers of parasitoid progeny were not affected by size of storage structures. In previous studies, the numbers of adult progeny of *H. hebetor* were not different when the sizes of rearing containers were varied [20,44] and the results were consistent when *P. interpunctella*, *E. kuehniella*, or the combination of both species were offered to *H. hebetor* [51].

### 6.5. Effect of Semiochemicals on the Recruitment of Habrobracon hebetor and Pteromalus cerealellae to Storage Containers

The potential role of semiochemicals in the location of hosts or host habitats by parasitoids of the families Braconidae and Pteromalidae have been studied [33,62]. Volatile compounds emitted by the host, or host-related odor sources, such as feces, frass, debris, host habitat, or food, were identified as the stimuli eliciting the strongest attractions to female parasitoids of *H. hebetor* and *P. cerealellae* [33,62,63,64]. Stimuli have been identified to elicit short- and long-range responses by the parasitoids [62]. The stimuli that elicit long-range responses recruit parasitoids to the habitat of the hosts, while short-range responses lead the parasitoids to the host. Some authors hypothesize that integrating these semiochemicals with parasitoid mass release programs could enhance parasitoid efficiency [62].

### 6.6. Effect of Age on the Fecundity of Habrobracon hebetor and Pteromalus cerealellae

Parasitoid age is a critical factor influencing the reproductive performances of *H. hebetor* [65]. Older females of *H. hebetor* (10 days old) have been shown to produce fewer progeny when reared on *E. kuehniella* and *G. mellonella*; progeny reduction when reared on *E. kuehniella* or *G. mellonella* was 34% and 26.7%, respectively [65]. Male-biases of parasitoid offspring became more pronounced with older parasitoids, irrespective of whether they were reared on *E. kuehniella* or *G. mellonella* [65].

With respect to *P. cerealellae*, progeny production has been shown to be significantly affected by the parasitoid age [55]. In a study by Onagbola et al., provisioning with supplemental food, such as glucose for *P. cerealellae* females, averted the effect of aging on oviposition, as 16–20-day-old female parasitoids that were fed on sugar solution produced significantly more progeny than starved females of the same age group [55].

### 6.7. Influence of Temperature on Habrobracon hebetor and Pteromalus cerealellae

Host parasitism and life history traits of *H. hebetor* are affected by abiotic stresses including temperature, moisture, and light [66,67]. Foraging behavior and reproductive performance of *H. hebetor* are affected by temperature inside storage facilities [68,69,70]. Egg production by the Braconid ectoparasitoid has been shown to increase with temperature and maximum fecundity occurred at 25 °C [68], while egg laying capacity was observed to decline at cold temperatures of 3 and 5 °C [71,72,73]. In addition, cooler storage temperatures have the potential to decrease parasitism, resulting in a decrease in host mortality [72]. Though the developmental time of immature stages of *H. hebetor* have been shown to decrease with increases in temperature [72], the lower threshold observed for development was between 11–12 °C [74]. The larval stages of *H. hebetor* are very sensitive to temperature. *H. hebetor* can survive and complete its life cycle within a temperature range of 15–40 °C [75]. The sex ratio of *H. hebetor* is affected by temperature and exposure time [69]. For instance, the sex ratio of *H. hebetor* offspring skewed towards males when parents were kept at 3 or 5 °C for 4 or 3 weeks, respectively [72].

## 7. Combined Application of Parasitoids and Other Biocontrol Agents

### 7.1. Combination of Parasitoid Species

In a study by Castañé et al., *Habrobracon hebetor* was able to affect 30% and 40% mortality of *E. kuehniella* and *P. interpunctella* in small storage experiments; interestingly, combining *H. hebetor* and *Venturia canescens* (Gravenhorst) (Hymenoptera: Ichneumonidae) against the same hosts in the same system did not generate host mortalities that were significantly higher than the application of *H. hebetor* alone [61].

### 7.2. Combination of Habrobracon hebetor and Bacillus thuringiensis

A study showed that combined application of *H. hebetor* and *Bacillus thuringiensis* (Bt) caused 86% mortality of the exposed *P. interpunctella* larvae, while deploying *H. hebetor* and Bt separately resulted in about 35% and 42% mortality, respectively [76]. It has been reported that the efficiency of *H. hebetor* as a parasitoid increased when the wasp was cultured with Bt [77]. For instance, 64%, 66%, and 73% mortality rates of *Corcyra cephalonica* (Lepidoptera: Gelleridae) (Stainton) larvae were obtained when *H. hebetor* was reared on a diet fortified with LC_25_, LC_10_, and LC_50_ of Bt, respectively. Conversely, wasps reared without Bt resulted in 37% mortality of host larvae [77]. In addition, synergy has been demonstrated between Bt and *H. hebetor* in the management of the field pest *Sodoptera littoralis* Boisd (Lepidoptera: Noctuidae) [78]. However, interaction between the wasp and Bt reduced the oviposition and progeny size of the parasitoid [73].

### 7.3. Habrobracon hebetor and Entomopathogenic Nematodes (EPNs)

Entomopathogenic nematodes of the families of Steinernematidae and Heterorhabditidae have been studied extensively for the management of a wide range of stored product pests [79]. Entomopathogens have the potential to be incorporated into biological control strategies of post-harvest IPM programs. Nematodes parasitize host larvae through their mutualistic relationship with bacteria (*Xenorhabdus* for Steinernematidae and *Photorhabdus* for Heterorhabditidae) that inhabit the intestine of the nematodes [80]. The infective juvenile (IJ) nematodes enter the hemocoel of the hosts through natural apertures including the mouth, anus, or spiracles and release pathogenic bacteria that multiply and kill the hosts within 48 h [80].

Larvae of *P. interpunctella* are highly susceptible to the combined application of *Heterorhabditis indica* IJs and the parasitoid *H. hebetor.* The interaction between the two has been reported to result in either additive or synergistic increases in the mortality of *P. interpunctella* [80]. Nematode–parasitoid interaction had no significant effect on the survival of adult and pupal parasitoids but affected the survival of the parasitoid larvae, suggesting that proper timing of the application of IJs of EPN and parasitoid release could circumvent the impact the nematodes could have on the larvae of parasitoids [80]. Moreover, it has also been noted that other parasitoids have been integrated with entomopathogenic nematodes for successful management of post-harvest pests [81].

### 7.4. Integration of Habrobracon hebetor with Entomopathogenic Fungi

Entomopathogenic fungi have the potential to contribute significantly to the suppression of stored insect pests. These fungi have also been investigated for integration with parasitoids [82,83,84]. Strains of *Beauveria bassiana* and *Metarhizium anisopliae* have been found to be pathogenic to larvae of *H. hebetor*, but not the pupae [84]. However, some isolates of *B. bassiana* (EUT105) and *M. anisopliae* (M-396) did not show adverse effects on the larval and pupal stages of *H. hebetor* [84]. Stinging by *H. hebetor* has been shown to increase the susceptibility of *G. mellonella* to *B. bassiana,* because the *H. hebetor* attack increased the conidial germination of *B. bassiana* on host larvae resulting in reduced phenoloxidase (PO) activity and hemocyte encapsulation rate in *G. mellonella* larvae [85,86]. These entomopathogenic fungi strains could therefore be integrated with parasitic wasps in the management of stored product pests.

## 8. Commercial Applications of Parasitoids and Integration with Other Natural Enemies

A few studies have demonstrated the potential of parasitoid wasps in the management of post-harvest pests in commercial warehouses. For example, *H. hebetor* occurs naturally and was found with other hymenopteran wasps on *E. kuehniella*-infested wheat in Italy and was also reported in fig storage warehouses in California and Greece [87,88,89]. The use of *H. hebetor* with *Trichogramma evanescens* Westwood (Hymenoptera: Trichogrammatidae) against *E. kuehniella* and *P. interpunctella* in organic bakeries and mills in Germany and Austria has been demonstrated to be effective [90]. The combined use of *H. hebetor* and mating disruption (MD) to manage *P. interpunctella* populations in a chocolate factory (about 2000 m^2^ area and 5 m height) decreased moths captured on traps [91]. In a peanut stock warehouse, the release of *H. hebetor* alone resulted in the 66.1% and 97.3% reduction of *P. interpunctella* and *C. cautella,* respectively [92]. Combining *H. hebetor* with *Trichogramma pretiosum* increased mortality of *P. interpunctella* to 84.0% and that of *C. cautella* to 98.0% [92].

## 9. Conclusions

Extensive information is currently available regarding foraging behavior and functional responses of parasitoids to facilitate implementation of bio-rational integrated pest management strategies in post-harvest systems. These parasitoids should be deployed in the management of post-harvest pests in the organic food industries, especially when a few insect fragments could be tolerated. The integration of parasitoids and complimentary IPM tools can be deployed in the management of residual populations of stored product pests in warehouses and other grain or processed food storage structures. *H. hebetor* and *P. cerealellae* are cosmopolitan in distribution and well suited to a range of climatic and environmental conditions. Their integration into pest management can and should be more broadly implemented.
